# Ca^2+^/calmodulin and protein kinase C (PKC) reverse the vesicle fusion arrest by unmasking PIP_2_

**DOI:** 10.1126/sciadv.adr9859

**Published:** 2025-02-26

**Authors:** Houda Yasmine Ali Moussa, Kyung Chul Shin, Yongsoo Park

**Affiliations:** ^1^ Neurological Disorders Research Center, Qatar Biomedical Research Institute (QBRI), Hamad Bin Khalifa University (HBKU), Qatar Foundation, Doha, Qatar.; ^2^ College of Health & Life Sciences (CHLS), Hamad Bin Khalifa University (HBKU), Qatar Foundation, Doha, Qatar.

## Abstract

Vesicle fusion is a key process in cellular communication and membrane trafficking. Soluble *N*-ethylmaleimide–sensitive factor attachment protein receptor (SNARE) proteins drive vesicle fusion, and SNARE proteins seem to be partially assembled before fusion occurs. However, the molecular mechanisms of the vesicle fusion arrest and how vesicle fusion is rescued from the arrest remain not fully understood. We have previously shown that as a lipid catalyst, phosphatidylinositol 4,5-bisphosphate (PIP2) electrostatically triggers vesicle fusion by lowering the hydration energy, and masking PIP2 arrests vesicle fusion in a state of the partial SNARE assembly. In this study, we show that calmodulin and protein kinase C–epsilon unmask PIP2 through the dissociation of myristoylated alanine-rich C-kinase substrate from membranes and, thus, rescue basal fusion and potentiate synaptotagmin-1–mediated Ca^2+^-dependent vesicle fusion. We provide the model in which the arrest of vesicle fusion can be rescued by the unmasking of PIP2, a lipid catalyst for fusion.

## Teaser

By lowering the hydration energy barrier, PIP_2_ is the lipid catalyst for vesicle fusion.

## INTRODUCTION

Neurotransmitter release and vesicle fusion are the processes on how neurons and neuroendocrine cells communicate with each other (*1*–*3*). Different types of vesicles store different types of neurotransmitters; synaptic vesicles (SVs) contain classical neurotransmitters, while large dense-core vesicles (LDCVs) carry amines, neuropeptides, and hormones (*4*–*6*). Vesicle fusion is driven by soluble *N*-ethylmaleimide–sensitive factor attachment protein receptor (SNARE) proteins (*1*, *2*) and synaptotagmin-1 is a Ca^2+^ sensor for fast Ca^2+^-dependent vesicle fusion (*6*). The time delay from presynapse to postsynapse for an electrical signal to travel across the synapse is 100 to 200 μs at 37°C (*7*, *8*); this observation suggests that ultrafast vesicle fusion might happen on the order of a few tenths of microseconds at physiological temperature after Ca^2+^ influx in the presynapse (*7*, *8*). In addition, SVs are tightly docked 1 to 5 nm from the active zone at synapses to be ready for fusion (*9*, *10*). Therefore, the SNARE complex seems to be partially preassembled before fusion occurs and vesicle fusion needs to be arrested in a vesicle docking state.

Recently, we have reported that phosphatidylinositol 4,5-bisphosphate (PIP2) with a −4 net charge at pH 7 (*11*) is the lipid catalyst for vesicle fusion by lowering the hydration energy barrier when vesicles are tightly docked by partial SNARE assembly (*12*). Masking PIP2 by myristoylated alanine-rich C-kinase substrate (MARCKS) arrests vesicle fusion in a state of the partial SNARE assembly and tight docking (*12*). However, the molecular mechanisms on how the vesicle fusion arrest is rescued remain unclear.

Here, we show that Ca^2+^/calmodulin (CaM) and protein kinase C (PKC)–epsilon rescue vesicle fusion from the arrest by dissociating the effector domain (ED) of MARCKS from membranes and thus unmasking PIP2. Synaptotagmin-1 is the primary Ca^2+^ sensor for fast exocytosis by inducing local membrane bending that lowers energy barrier for fusion (*13*). CaM is also considered as an additional Ca^2+^ sensor for vesicle fusion (*14*), e.g., vacuoles in yeast (*15*), endosomes (*16*), cortical granules in sea urchin eggs (*17*), LDCVs (*18*–*21*), and SVs (*22*–*25*), although the contributing function of Ca^2+^/CaM on vesicle fusion as a Ca^2+^ sensor is controversial. Using interdisciplinary approaches including the in vitro reconstitution of native vesicle fusion in a physiological context and amperometry to monitor exocytosis in real time, we address the mechanisms how Ca^2+^/CaM and PKC regulate both Ca^2+^-dependent and Ca^2+^-independent vesicle fusion by unmasking PIP2.

### Ca^2+^/CaM rescues LDCV fusion by unmasking PIP_2_

**Fig. 1.**
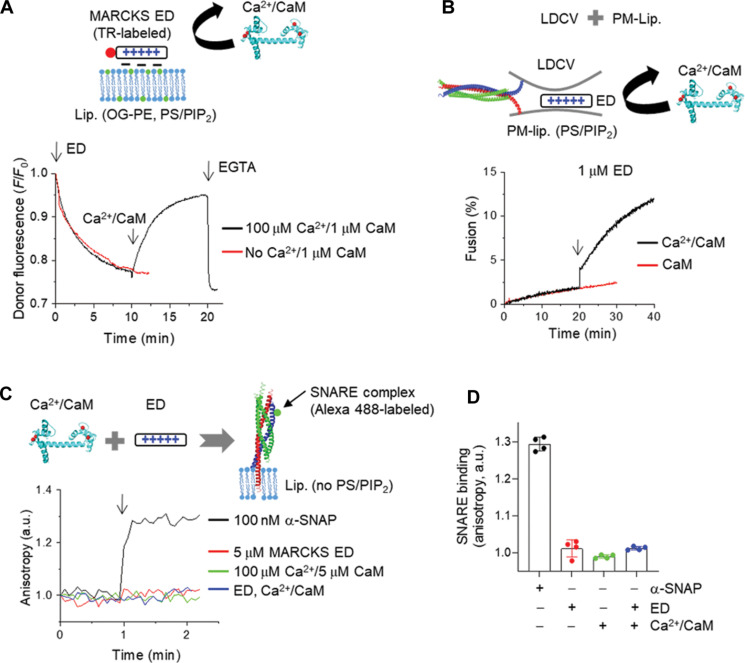
Ca^2+^/CaM rescues LDCV fusion by unmasking PIP_2_. (**A**) Membrane association of MARCKS was monitored using FRET measurement. The effector domain (ED) of MARCKS (amino acids 151 to 175) was labeled with BODIPY TR (red dot) and liposomes (lip., protein-free) incorporated Oregon Green (OG) as an acceptor and a donor dye, respectively. Lipid composition of liposomes: 45% PC, 13.5% PE, 1.5% OG 488-DHPE, 10% PS, 25% Chol, 4% PI, and 1% PIP2. MARCKS ED was associated with liposomes and then 100 μM free Ca^2+^ along with 1 μM CaM induced the dissociation of MARCKS ED from liposomes containing PS/PIP2. EGTA (1 mM) reversed the membrane dissociation of ED by chelating Ca^2+^. (**B**) In vitro reconstitution of vesicle fusion using a lipid-mixing assay. ED was preincubated with LDCVs and completely blocked basal LDCV fusion with PM-lip by masking PIP2. Sequential treatment with Ca^2+^/CaM (indicated by the arrow) rescued LDCV fusion from this arrest. (**C** and **D**) Interaction of the SNARE complex with MARCKS ED and/or Ca^2+^/CaM was monitored using anisotropy measurement. The stabilized Q-SNARE complex containing VAMP-2 (residues 49–96) labeled with Alexa Fluor 488 (Alexa 488–labeled VAMP-2) was incorporated in liposomes that contained no PS/PIP2: 60% PC, 15% PE, and 25% Chol. (D) Data are means ± SD from four independent experiments. Physiological ionic strength with 1 mM MgCl2/3 mM ATP was used in all experiments, unless stated otherwise; ATP removes contaminating Ca^2+^ as a Ca^2+^ chelator (*34*) and disrupts nonspecific weak interaction of SNARE-synaptotagmin-1 (*31*). a.u., arbitrary units.

The ED (25 amino acids) of MARCKS with 13 basic residues electrostatically masks PIP2 (*26*–*28*). We have previously reported that MARCKS ED arrests vesicle fusion in a state of tight docking and partial SNARE assembly by masking PIP2, which is a lipid catalyst for vesicle fusion (*12*). Native vesicles such as LDCVs and SVs are essential for the in vitro reconstitution of physiological vesicle fusion (*12*, *13*, *29*–*33*). Given that adenosine triphosphate (ATP) removes contaminating Ca^2+^ as a Ca^2+^ chelator (*34*) and disrupts nonspecific weak interaction of SNARE-synaptotagmin-1 (*31*) and the cis-interaction of synaptotagmin-1 (*29*, *34*), physiological ionic strength with 1 mM MgCl2/3 mM ATP is critical for the reconstitution of vesicle fusion in a physiological context; MgCl2/ATP were used in all experiments, unless stated otherwise. Because the reversible membrane association and dissociation of MARCKS ED are regulated by Ca^2+^/CaM (*35*–*37*), we tested whether Ca^2+^/CaM dissociates MARCKS ED from PIP2-containing membranes by using fluorescence resonance energy transfer (FRET) measurement (Fig. 1A). MARCKS ED was labeled with BODIPY TR at the N terminus as an acceptor and Oregon Green–PE was incorporated in protein-free liposomes as a donor dye. Membrane binding of BODIPY TR–labeled MARCKS ED led to quenching of donor fluorescence (Fig. 1A). MARCKS ED initially interacted with liposomes that contain PS/PIP2 (Fig. 1A), as reported previously (*12*). The treatment of 100 μM free Ca^2+^ along with 1 μM CaM induced the dissociation of MARCKS ED from liposomes containing PS/PIP2. This dissociation of ED was subsequently reversed by the addition of 1 mM EGTA, which chelated Ca^2+^, confirming the dynamic and reversible membrane dissociation of ED by Ca^2+^/CaM (Fig. 1A). CaM alone interacted with ED (fig. S1A), but both Ca^2+^ and CaM were essential for the displacement of ED from PIP2, as Ca^2+^/CaM shifted the equilibrium toward the dissociated state (*38*), thereby unmasking PIP2 (Fig. 1A). Next, we investigated whether Ca^2+^/CaM rescues vesicle fusion by unmasking PIP2. In our reconstitution system (*12*, *13*, *29*–*33*), native vesicles readily fuse with the plasma membrane-mimicking liposomes (PM-liposomes) that contain the stabilized Q-SNARE complex of syntaxin-1A (amino acids 183 to 288) and SNAP-25A in a 1:1 ratio by a C-terminal fragment of VAMP-2 (*39*) (Fig. 1B). ED (1 μM) completely arrests LDCV fusion in a state of vesicle docking through the partially zippered SNARE complex (*12*); ED disrupted LDCV basal fusion by masking PIP2, which is an electrostatic catalyst for vesicle fusion. We observed that basal LDCV fusion arrest by 1 μM ED was reversed and rescued by Ca^2+^/CaM that triggered LDCV fusion like a step increase; CaM alone was ineffective (Fig. 1B), supporting that Ca^2+^/CaM rescued LDCV fusion by unmasking PIP2. We confirmed no interactions of the SNARE complex with MARCKS ED and/or Ca^2+^/CaM by using anisotropy (Fig. 1, C and D). The VAMP-2 (residues 49–96) labeled with Alexa Fluor 488 (Alexa 488–labeled VAMP-2) is incorporated in the stabilized Q-SNARE complex in PM-liposomes that contained no PS/PIP2 (Fig. 1C). As a control, α-soluble NSF attachment protein (α-SNAP), a cofactor for SNARE disassembly, bound to the SNARE complex (Fig. 1, C and D). Altogether, MARCKS and Ca^2+^/CaM regulated vesicle fusion by masking and unmasking PIP2, not by interacting with the SNARE complex.

### MARCKS prevents C2AB membrane binding by masking PIP_2_

**Fig. 2.**
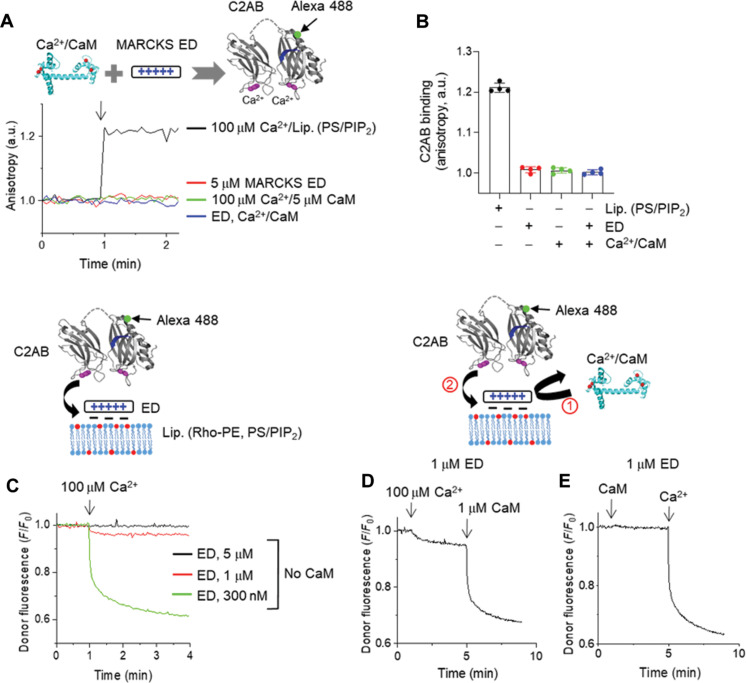
Unmasking PIP_2_ by Ca^2+^/CaM leads to synaptotagmin-1 membrane binding. (**A** and **B**) Anisotropy was carried out to monitor the interaction of the C2AB domain of synaptotagmin-1 with MARCKS ED and/or Ca^2+^/CaM. The C2AB domain (residues 97–421) was labeled with Alexa Fluor 488 at S342C (green dot). No liposomes were included. Liposomes containing PS/PIP2 with 100 μM Ca^2+^, a positive control. Data are means ± SD from four independent experiments. (**C** to **E**) Monitoring the membrane binding of the C2AB domain using FRET measurement. The C2AB domain was labeled with Alexa Fluor 488 at S342C (green dot) as a donor dye, and liposomes (Lip.) incorporated rhodamine (Rho)–PE (red dot) as an acceptor dye. Lipid composition of liposomes for FRET: protein-free, 45% PC, 13.5% PE, 1.5% Rho-PE, 10% PS, 25% Chol, 4% PI, and 1% PIP2. (C) MARCKS ED interfered with the C2AB membrane binding by masking PIP2. Free Ca^2+^ (100 μM) was applied to evoke the C2AB membrane binding. [(D) and (E)] In the presence of 1 μM MARCKS ED that blocks the C2AB membrane binding, 100 μM free Ca^2+^ and 1 μM CaM were sequentially applied to (i) dissociate ED from membranes, thus (ii) rescuing the C2AB membrane binding. FRET was normalized as *F*/*F*0, where *F*0 is the initial value of the donor fluorescence intensity. Physiological ionic strength with 1 mM MgCl2/3 mM ATP was used in all experiments.

PIP2 is also required for Ca^2+^-triggered fusion of native vesicles by inducing the trans-interaction of synaptotagmin-1 that leads to membrane deformation and bending of the plasma membrane; in the absence of PIP2 in PM-liposomes, Ca^2+^-dependent fusion of native vesicles does not occur (*29*, *31*, *34*). Therefore, we investigated whether PIP2-masking MARCKS ED reduces synaptotagmin-1 binding to PIP2-containing membranes. As a control, using anisotropy measurement, we confirmed no interaction of the C2AB domain with MARCKS ED, Ca^2+^/CaM, or both (Fig. 2, A and B). The C2AB domain (residues 97–421) of synaptotagmin-1 was labeled with Alexa Fluor 488 at S342C as a donor, and liposomes (Lip., protein-free, 10% PS/1% PIP2 included) were labeled with rhodamine (Rho)–PE as an acceptor (Fig. 2, C to E). The C2AB binding to liposomes was monitored using FRET between the C2AB domain (Alexa Fluor 488) and Rho-labeled liposomes (Fig. 2, C to E). In the absence of ED, 100 μM free Ca^2+^ caused robust membrane binding of the C2AB domain, and Ca^2+^/CaM did not affect the C2AB binding to liposomes (fig. S2A), correlating with no interaction of the C2AB domain with Ca^2+^/CaM (Fig. 2, A and B). As expected, MARCKS ED prevented Ca^2+^-induced membrane binding of the C2AB domain in a dose-dependent manner by masking PIP2 (Fig. 2C). In the presence of 1 μM ED, Ca^2+^ and CaM were applied sequentially to rescue the C2AB membrane binding by dissociating ED and unmasking PIP2 (Fig. 2, D and E). The addition of 100 μM Ca^2+^ alone slightly induced C2AB binding, and then sequential treatment of Ca^2+^/CaM accelerated and rescued the C2AB membrane binding (Fig. 2D). CaM alone did not rescue the C2AB membrane binding, but Ca^2+^/CaM caused the membrane dissociation of ED (Fig. 2E), thereby rescuing the C2AB membrane binding by unmasking PIP2 (Fig. 2, D and E).

### Ca^2+^/CaM potentiates Ca^2+^-dependent vesicle fusion

**Fig. 3.**
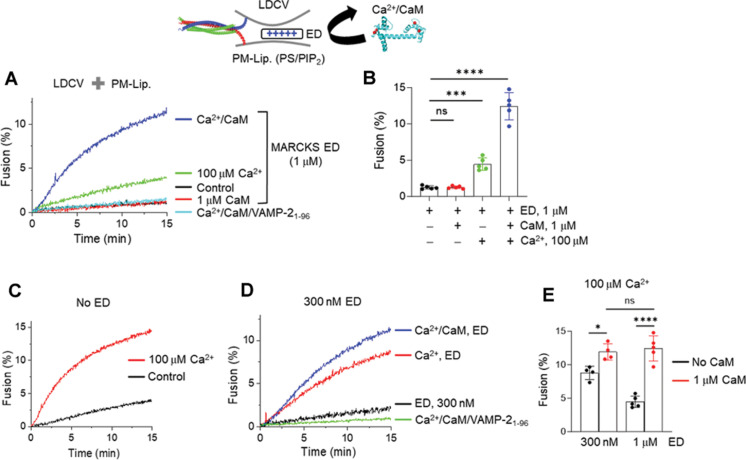
Ca^2+^/CaM rescues and triggers vesicle fusion arrested by MARCKS ED. (**A** to **E**) LDCV fusion with PM-liposomes using a lipid-mixing assay as in Fig. 1B. [(A) and (B)] Soluble VAMP-2 (residues 1–96), the cytoplasmic domain of VAMP-2 (amino acids 1 to 96), completely disrupted SNARE-dependent fusion. MARCKS ED (1 μM) completely inhibited LDCV basal fusion to the level of soluble VAMP-2. Free Ca^2+^ (100 μM) alone slightly recovered LDCV fusion, but Ca^2+^/CaM reversed LDCV fusion from the arrest. (C) No MARCKS ED was included. Ca^2+^ (100 μM) accelerated LDCV fusion. [(D) and (E)] In the presence of 300 nM MARCKS ED, Ca^2+^ alone accelerated LDCV fusion, and Ca^2+^/CaM potentiated Ca^2+^-dependent LDCV fusion. Data in (B) and (E) are means ± SD from four to five independent experiments. One-way ANOVA test with Bonferroni correction was used; *P < 0.05, ***P < 0.001, ****P < 0.0001. ns, not significant.

The data presented above suggest that MARCKS ED inhibits Ca^2+^-dependent membrane binding of the C2AB domain by masking PIP2, and Ca^2+^/CaM recovers the C2AB membrane binding by dissociating ED and unmasking PIP2 (Figs. 1 and 2). Next, we further tested whether Ca^2+^/CaM potentiates Ca^2+^-dependent vesicle fusion by unmasking PIP2. Addition of 1 μM ED completely inhibited LDCV basal fusion to the level of soluble VAMP-2 (residues 1–96) treatment, and 1 μM CaM without Ca^2+^ in the presence of ED was unable to rescue basal fusion (Fig. 3, A and B), because CaM without Ca^2+^ failed to dissociate ED (Fig. 1A). In the presence of 1 μM ED, but the absence of CaM, 100 μM Ca^2+^ alone slightly induced Ca^2+^-dependent vesicle fusion (Fig. 3, A and B), correlating with the weak C2AB binding to liposomes (100 μM Ca^2+^ and 1 μM ED, Fig. 2, C and D). Ca^2+^/CaM potentiated Ca^2+^-dependent vesicle fusion (Fig. 3, A and B); this result correlates with the ability of Ca^2+^/CaM to rescue the C2AB membrane binding by unmasking PIP2 (Fig. 2, D and E). Together, these results indicate that Ca^2+^/CaM unmasks PIP2 by dissociating MARCKS ED, and thereby potentiates Ca^2+^-dependent vesicle fusion, because the C2AB domain of synaptotagmin-1 requires PIP2 to trigger Ca^2+^-dependent fusion (*13*, *29*, *31*).

PIP2 actively interacts with partner proteins for various cellular functions (*40*); thus, free PIP2 is tightly regulated in cells. Therefore, we tested MARCKS ED at low concentrations, at which free PIP2 is available for basal fusion. In the absence of ED, LDCVs underwent robust basal fusion, and 100 μM Ca^2+^ accelerated LDCV fusion (Fig. 3C). The addition of 1 μM ED completely arrested basal LDCV fusion (Fig. 3, A and B). In the presence of 300 nM ED, basal fusion was slightly rescued (Fig. 3D) (*12*); the addition of Ca^2+^ alone increased LDCV fusion, but Ca^2+^ along with CaM potentiated Ca^2+^-dependent LDCV fusion by dissociating ED (Fig. 3, D and E). Increasing ED concentrations strongly inhibited Ca^2+^-dependent fusion, but Ca^2+^/CaM rescued it by unmasking PIP2 (Fig. 3E).

### Ca^2+^/CaM potentiates SV fusion by unmasking PIP_2_

**Fig. 4.**
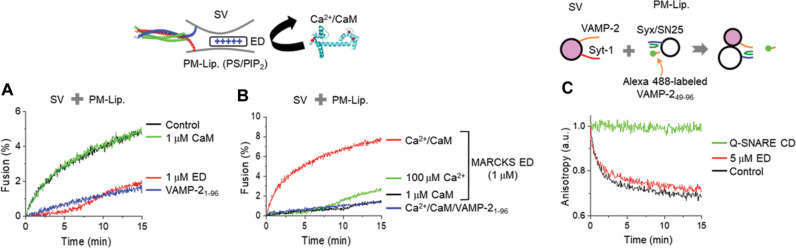
Ca^2+^/CaM reverses SV fusion from the arrest by unmasking PIP_2_. (**A** and **B**) SVs are used in a lipid-mixing assay to monitor SV fusion with PM-liposomes. (A) MARCKS ED (1 μM) completely inhibited SV fusion to the level of soluble VAMP-2, whereas CaM alone had little effect on SV fusion. (B) Ca^2+^ alone slightly induced SV fusion, and Ca^2+^/CaM rescued SV fusion from the arrest induced by PIP2-masking ED. (**C**) Monitoring SNARE assembly using fluorescence anisotropy. The stabilized Q-SNARE complex containing VAMP-2 (residues 49-96) labeled with Alexa Fluor 488 (Alexa 488–labeled VAMP-2) was incorporated in PM-liposomes. Displacement of Alexa 488–labeled VAMP-2 by endogenous VAMP-2 of native SVs represents SNARE assembly. Preincubation of the Q-SNARE cytoplasmic domain (CD) consisting of syntaxin-1A (amino acids 183 to 262) and SNAP-25A with SVs blocked SNARE assembly, and thus prevented dissociation of Alexa 488–labeled VAMP-2. Lipid composition for SNARE assembly: 45% PC, 15% PE, 10% PS, 25% Chol, 4% PI, and 1% PIP2. Anisotropy was normalized as *A*/*A*0, where *A*0 is the initial value of anisotropy.

We further tested SV fusion to obtain generalized conclusions that Ca^2+^/CaM potentiates Ca^2+^-dependent fusion by unmasking PIP2. The addition of 1 μM MARCKS ED completely inhibited SV basal fusion to the level of soluble VAMP-2 treatment by PIP2 masking (Fig. 4A), reminiscent of LDCV fusion (Fig. 3, A and B). In the absence of Ca^2+^, CaM had little effect on basal SV fusion (Fig. 4A). The addition of 100 μM Ca^2+^ in the presence of 1 μM ED slightly induced Ca^2+^-dependent vesicle fusion, but Ca^2+^/CaM potentiated Ca^2+^-dependent SV fusion by dissociating MARCKS ED and unmasking PIP2 (Fig. 4B); this process is consistent with LDCV fusion (Fig. 3). We tested whether CaM might synergize with synaptotagmin-1 to induce Ca^2+^-dependent vesicle fusion independently of MARCKS. In the absence of MARCKS ED, 100 μM Ca^2+^ accelerated SV fusion, but CaM had no effect on Ca^2+^-dependent SV fusion mediated by synaptotagmin-1 (fig. S3A), supporting that CaM has no synergistic effect with synaptotagmin-1, but potentiates Ca^2+^-dependent SV fusion by unmasking PIP2. Altogether, free PIP2, which is dynamically regulated by MARCKS and Ca^2+^/CaM, is the key to trigger both basal fusion and Ca^2+^-dependent fusion of native vesicles. As a control, we monitored SNARE assembly by using fluorescence anisotropy (Fig. 4C). The Alexa 488–labeled VAMP-2 (residues 49–96) fragment is displaced from the stabilized Q-SNARE complex as the full-length VAMP-2 zippers in the N-to-C terminal direction (*12*, *29*, *31*, *39*). The displacement of the Alexa 488–labeled VAMP-2 fragment by endogenous VAMP-2 of SVs causes a decrease in fluorescence anisotropy due to the rotational mobility of fluorophore (*29*, *30*). As expected, even in the presence of 5 μM ED that fully arrested basal SV fusion (Fig. 4A), SNARE zippering and assembly occurred, supporting that SV fusion is arrested in a state of docking through partial SNARE assembly (Fig. 4C).

### Unmasking of PIP_2_ by PKC induces Ca^2+^-independent fusion

**Fig. 5.**
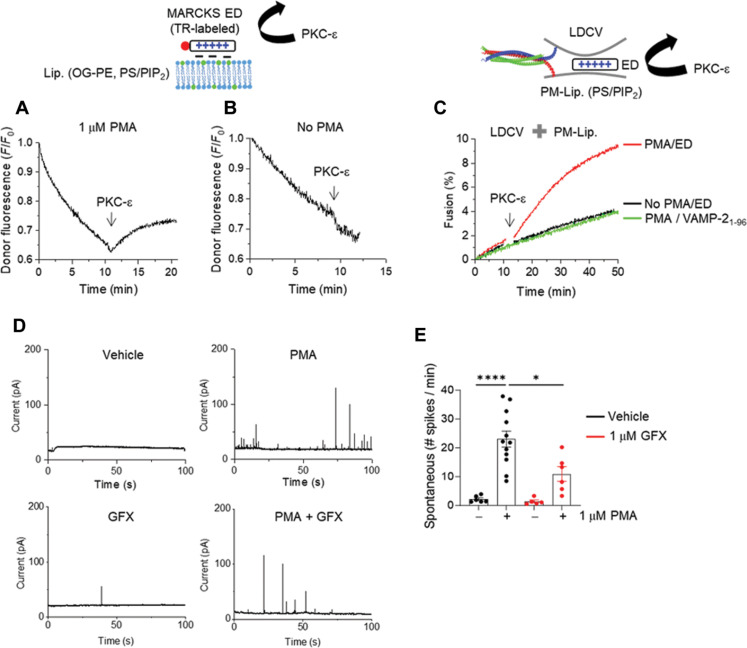
PKC-epsilon dissociates MARCKS ED and rescues vesicle fusion. (**A** and **B**) Monitoring membrane association and disassociation of MARCKS ED using FRET measurement as in Fig. 1A. PKC-epsilon (60 nM) was applied in the presence (A) or absence (B) of 1 μM PMA. 10% PS/1% PIP2 were included in liposomes. (**C**) LDCV fusion using a lipid-mixing assay. PKC-epsilon (60 nM) with 1 μM PMA recovered LDCV fusion from the arrest induced by 1 μM MARCKS ED. (**D** and **E**) Monitoring LDCV exocytosis in bovine chromaffin cells using amperometry. Shown are typical amperometric traces of chromaffin cells pretreated for 5 min with DMSO vehicle or 1 μM PMA in the presence or absence of 1 μM GFX. (E) Spontaneous LDCV exocytosis is presented as numbers of amperometric spikes per min. Data are means ± SEM from three independent experiments (*n* = 6, 12, 5, and 6). One-way ANOVA test with Bonferroni correction was used; *P < 0.05, ****P < 0.0001.

MARCKS electrostatically masks PIP2, and Ca^2+^/CaM unmasks PIP2 by dissociating MARCKS from PIP2-containing membranes (Fig. 2), thus leading to potentiation of Ca^2+^-dependent vesicle fusion (Figs. 3 and 4), because CaM displaces MARCKS from PIP2-containing membranes in a Ca^2+^-dependent manner (Fig. 1A). As a lipid catalyst for vesicle fusion, PIP2 is also essential and required for Ca^2+^-independent basal fusion of native vesicles (*12*). Basal fusion and some spontaneous releases in vivo are Ca^2+^ independent; thus, we hypothesized that Ca^2+^-independent membrane displacement of MARCKS ED can trigger spontaneous release and basal fusion. PKC drives the displacement of MARCKS from PIP2-containing membranes through phosphorylation, shifting the equilibrium toward dissociation (*27*, *41*). PKC-epsilon, an isoform of the PKC subfamily, is activated by diacylglycerol (DAG), but not by Ca^2+^ (*42*). In the final set of experiments, we tested whether the membrane dissociation of MARCKS ED by PKC-epsilon induces the basal fusion of LDCVs. We monitored the membrane dissociation of ED by using FRET as in Fig. 1A. PKC-epsilon dissociated ED only in the presence of phorbol 12-myristate 13-acetate (PMA), an analog of DAG and an activator of PKC (Fig. 5, A and B). The addition of 1 μM ED completely arrested LDCV fusion to the level of soluble VAMP-2, as shown in Fig. 3, A and B. Notably, the activation of PKC-epsilon rescued and restored basal LDCV fusion by dissociating ED (Fig. 5C). Next, we investigated the idea that PKC activation increases the spontaneous release of LDCVs in chromaffin cells. To test the PKC effect on basal fusion, we used amperometry to monitor spontaneous LDCV exocytosis in real time. Treatment with PMA increased the spontaneous release of LDCVs, which was reduced by GF109203X (GFX), an inhibitor of PKC (Fig. 5, D and E). Together, ED dissociation by PKC-epsilon triggers Ca^2+^-independent basal fusion by unmasking PIP2.

## DISCUSSION

The SNARE complex seems to be partially assembled before fusion occurs with the following reasons: (i) vesicle fusion is fast on the order of a few tenths of microseconds to submilliseconds depending on temperature (*7*, *8*); (ii) the SNARE motif is ~7 nm long (*43*) and the vesicle docking is tight, i.e., 1 to 5 nm distance between the vesicle and the plasma membrane (*9*, *10*); and (iii) the partial assembly of the SNARE complex allows multiple SNARE complexes to coordinate and contribute to fusion (*44*, *45*). Partial assembly ensures that multiple SNARE complexes are positioned correctly, ready to complete the final steps in a coordinated manner. 

Several models have been proposed to explain the SNARE clamping mechanism involving synaptotagmin-1 and complexin. However, most of these models are based on experiments conducted under low ionic strength conditions, which may not accurately reflect physiological environments (*6*). As a result, the mechanisms of SNARE clamping remain controversial (*6*). In addition, considering that SNARE assembly provides the necessary energy to overcome the barriers to fusion, but SNARE complexes are already preassembled in a tightly docked state before fusion, it remains unclear how this energy barrier is effectively overcome to facilitate vesicle fusion.

**Fig. 6.**
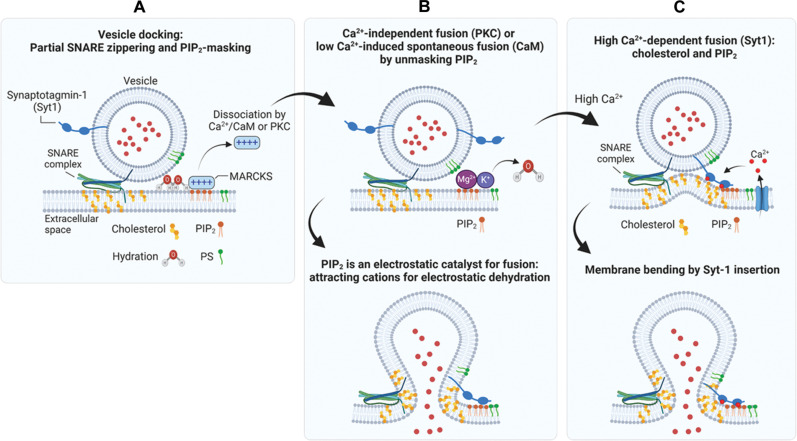
Schematic diagram of the proposed model for Ca^2+^-independent basal fusion and Ca^2+^-dependent evoked vesicle fusion. (**A**) Partial SNARE assembly induces vesicle docking. PIP2-masking proteins, e.g., MARCKS, arrest Ca^2+^-independent basal fusion in a state of vesicle docking. Other PIP2-binding proteins can arrest basal fusion. (**B**) For Ca^2+^-independent basal fusion and low Ca^2+^-induced spontaneous vesicle fusion, PKC and Ca^2+^/CaM unmask PIP2 by dissociating MARCKS. PIP2 is a lipid catalyst for vesicle fusion by inducing electrostatic dehydration; i.e., PIP2 attract cations that reduce the repulsion of two membranes and repel water molecules from the membrane surface, thus lowering the hydration energy barrier. CaM is a high-affinity Ca^2+^ sensor in the range of 1 to 10 μM Ca^2+^. (**C**) For high Ca^2+^-dependent evoked vesicle fusion, PIP2 causes the trans-interaction of synaptotagmin-1 with the plasma membrane and synaptotagmin-1 triggers vesicle fusion through membrane bending, which is strengthened by cholesterol. Ca^2+^/CaM and PKC rescue basal fusion from the arrest and potentiate Ca^2+^-dependent vesicle fusion. Created with BioRender.com.

We proposed the model that PIP2, as a lipid catalyst for vesicle fusion, electrostatically triggers vesicle fusion by lowering the hydration energy barrier when vesicles are tightly docked through partial SNARE assembly (*12*). PIP2 attracts cations that reduce repulsion between two membranes and repel water molecules from the membrane surface (*12*). Therefore, we propose a term called electrostatic dehydration, which describes the process in which the binding of cations to negatively charged lipids leads to a reduction in the hydration shell around the lipid headgroups (*12*). In this study, PIP2 masking by MARCKS arrests vesicle fusion in a tight docking state by partial SNARE assembly (Fig. 1). Ca^2+^/CaM dissociates MARCKS, then unmasks PIP2, thereby rescuing vesicle fusion from the arrest. Ca^2+^/CaM potentiates synaptotagmin-1–mediated Ca^2+^-dependent vesicle fusion (Figs. 3 and 4). MARCKS can also be dissociated to unmask PIP2 independently of Ca^2+^ by activating PKC-epsilon, thereby triggering Ca^2+^-independent basal fusion (Fig. 5). Together, PIP2 is an electrostatic catalyst required for vesicle fusion (Fig. 6).

For Ca^2+^-independent fusion, free PIP2 electrostatically triggers vesicle fusion by attracting cations that eventually reduce the hydration energy barrier, i.e., electrostatic dehydration (*12*) (Fig. 6, A and B). CaM, a high-affinity Ca^2+^ sensor in the range of 1 to 10 μM Ca^2+^, induces low Ca^2+^-induced spontaneous fusion by unmasking PIP2 (Fig. 6, A and B). Synaptotagmin-1 is responsible for high Ca^2+^-dependent fusion, as a low-affinity Ca^2+^ sensor in the range of 10 to 100 μM Ca^2+^ (Fig. 6C). PIP2 causes the trans-interaction of synaptotagmin-1 with the plasma membrane (*29*, *31*), and synaptotagmin-1 triggers vesicle fusion by inducing membrane bending, which is strengthened by cholesterol; i.e., no Ca^2+^ effect in triggering vesicle fusion is observed in the absence of cholesterol in the plasma membrane (*13*) (Fig. 6C). Ca^2+^ also fails to trigger synaptotagmin-1–mediated Ca^2+^-dependent fusion when PIP2 is removed from PM-liposomes (*29*), and PIP2 critically regulates the Ca^2+^ sensitivity and Ca^2+^ cooperativity of synaptotagmin-1 (*34*), indicating that PIP2 is essential for both Ca^2+^-dependent and Ca^2+^-independent vesicle fusion.

The arrest and clamping of SNARE assembly during vesicle docking and priming have been hypothesized, although the proteins proposed to cause this arrest, such as complexin and synaptotagmin-1, remain unclear (*6*). Synaptotagmin-1 is one of the candidates for SNARE clamping factors, but synaptotagmin-1–SNAREs interaction is observed at low ionic strength and Mg^2+^/ATP completely disrupts this weak synaptotagmin-1–SNAREs interaction by the charge shielding effect (*31*, *46*), arguing against synaptotagmin-1 as a SNARE clamping factor. Complexin I/II was proposed to clamp full SNARE zippering by binding to the SNARE complex (*47*, *48*). However, spontaneous vesicle fusion remains unchanged or is diminished in complexin-deficient mammalian neurons (*49*–*53*), questioning complexin as a SNARE clamping factor.

In addition, α-SNAP binds to the Q-SNARE complexes and interferes with full SNARE zippering, thus slowing down fusion and causing tight docking as a result of partially assembled SNARE proteins (*30*). The SNARE complex is disassembled by the AAA+ ATPase *N*-ethylmaleimide–sensitive factor (NSF) and a cofactor, α-SNAP. α-SNAP first binds to the SNARE complex with a high binding affinity, and then recruits NSF, thereby catalyzing SNARE disassembly in an ATP-dependent manner (*54*). α-SNAP that binds to the Q-SNARE complexes induces partial SNARE zippering in a state of tight docking of LDCVs (*30*). α-SNAP markedly inhibits LDCV (~150 nm in diameter) fusion within 5 min after fusion reaction, but does not affect SV (~45 nm in diameter) fusion, because high curvature of small SVs is likely to overcome the inhibitory effect of α-SNAP (*30*). The curvature of vesicle membrane affects the energy landscape during fusion reaction, so arresting SNARE assembly of small SVs might be energetically challenging (*30*). Altogether, SNARE zippering and vesicle fusion are rarely halted or arrested by SNARE-interacting proteins in a tightly docked state.

Our data show that vesicle fusion depends on dynamic regulation of free PIP2 by CaM and PKC. In glutamatergic neurons, CaM is involved in Ca^2+^-dependent spontaneous release (*55*), supporting our model that Ca^2+^/CaM increases basal fusion by unmasking PIP2. Phorbol esters increase Ca^2+^-independent spontaneous release through Munc13 and PKC (*56*) in inhibitory and excitatory synapses (*57*–*59*), again supporting that PKC-epsilon can induce basal fusion in a Ca^2+^-independent manner by unmasking PIP2 (Fig. 5). Our data suggest that PKC increases spontaneous release, and Ca^2+^/CaM potentiates Ca^2+^-dependent vesicle fusion by unmasking PIP2. PIP2 constitutes approximately 1% of the total phospholipids in the plasma membrane (*27*). However, not all of the PIP2 present in the membrane is free. Cytoskeletal proteins, ion channels, and membrane-associated proteins bind to PIP2, thereby reducing the pool of free PIP2 (*27*). Determining the dynamic fraction of free PIP2 in living cells can be quite challenging, but MARCKS sequesters and masks substantial amounts of PIP2 within lateral membrane domains (*27*). CaM and PKC displace MARCKS by shifting the equilibrium toward the dissociated state, thereby unmasking PIP2 and triggering cellular signaling processes (*38*, *41*, *60*). Free PIP2 availability is essential for regulating ion channel activity, a key factor in long-term potentiation and long-term depression, and the fundamental processes underlying synaptic plasticity (*61*).

MARCKS is a highly abundant protein, particularly in the brain, constituting approximately 0.2% of total soluble protein (*62*), which corresponds to a concentration of 1 to 10 μM in neurons (*28*). This is comparable to the concentration of PIP2 (*27*, *63*). Given that MARCKS inhibits Ca^2+^-independent LDCV fusion in a dose-dependent manner, with an IC50 (median inhibitory concentration) of 127.6 nM and that 1 μM ED completely blocked basal fusion in our previous study (*12*), the MARCKS concentrations used in our experiments (less than 1 μM) are consistent with physiological levels. Similarly, CaM constitutes at least 0.1% of total cellular protein, corresponding to 1 to 10 μM (*64*, *65*). Therefore, 1 μM CaM used in our study falls within the physiological concentration range. PKC enzymes are typically present in cells at concentrations of 0.1 to 1 μM (*66*), validating that 60 nM of PKC-epsilon used in our study appropriately simulates physiological activity.

In summary, we present the model and paradigm that PIP2 is the lipid catalyst for vesicle fusion by lowering the hydration energy barrier and is required for both Ca^2+^-dependent and Ca^2+^-independent vesicle fusion (Fig. 6). It remains for further study to investigate whether other PIP2-binding proteins can inhibit spontaneous vesicle fusion by masking PIP2. This emerging paradigm, suggesting PIP2 as the fusion catalyst, will advance our understanding of the underlying mechanisms of the vesicle fusion arrest.

## MATERIALS AND METHODS

### Materials

ATP disodium salt was from Sigma-Aldrich (catalog no. A2383). Alexa Fluor 488 C5 maleimide (catalog no. A10254) and BODIPY TR-X succinimidyl ester (catalog no. D6116) were purchased from Thermo Fisher Scientific. The MARCKS ED (residues 151 to 175 of bovine MARCKS, termed MARCKS ED) consisting of KKKKKRFSFKKSFKLSGFSFKKNKK was synthesized by GenScript (Piscataway, NJ). Recombinant PKC-epsilon (catalog no. SRP5067), recombinant bovine CaM (catalog no. C4874), PMA (catalog no. P8139), and GFX (catalog no. B6292) were purchased from Sigma-Aldrich. All lipids were purchased from Avanti Polar lipids except Oregon Green 488 1,2-dihexadecanoyl-*sn*-glycero-3-phosphoethanolamine (Oregon Green 488 DHPE) (catalog no. O12650, Thermo Fisher Scientific).

### Purification of LDCVs and SVs

LDCVs, also known as chromaffin granules, were purified from bovine adrenal medullae by using continuous sucrose gradient and resuspended with fusion buffer containing 120 mM K-glutamate, 20 mM K-acetate, and 20 mM Hepes-KOH, pH 7.4, as described previously (*33*). Briefly, fresh bovine adrenal glands were obtained from a local slaughterhouse. The cortex and fat were removed, then the medullae were minced with scissors in 300 mM sucrose buffer (300 mM sucrose and 20 mM Hepes, pH 7.4, adjusted with KOH), and homogenized using a homogenizer. After centrifugation at 1000*g* for 15 min at 4°C, the pellet containing nuclei and cell debris (P1) was discarded. The supernatant (S1) was further centrifuged (12,000*g*, 15 min, 4°C), then subjected to an additional cycle of resuspension and centrifugation as a washing step. The resulting pellet (P2, crude LDCV fraction) was resuspended in 300 mM sucrose buffer and loaded on top of a continuous sucrose gradient (from 300 mM to 1.9 M) to remove other contaminants including mitochondria. LDCVs were collected from the pellet after centrifugation at 110,000*g* for 60 min in a Beckman SW 41 Ti rotor, then resuspended with the buffer (120 mM K-glutamate, 20 mM K-acetate, and 20 mM Hepes-KOH, pH 7.4). SVs from mouse brains were purified as described previously (*67*). Briefly, the brains were homogenized in homogenization buffer supplemented with protease inhibitors, using a homogenizer. The homogenate was centrifuged for 10 min at 1000*g*, and the resulting supernatant was further centrifuged (15 min, 15,000*g*, 4°C). The synaptosome pellet was lysed by adding ice-cold water, followed by centrifugation (25 min, 48,000*g*, at 4°C). The resulting supernatant was overlaid onto a 0.7 M sucrose cushion and centrifuged for 1 hour at 133,000*g*. The pellet was resuspended in the buffer (120 mM K-glutamate, 20 mM K-acetate, and 20 mM Hepes-KOH, pH 7.4).

### Protein purification

All SNARE and synaptotagmin-1 constructs based on *Rattus norvegicus* sequences were expressed in *Escherichia coli* strain BL21 (DE3) and purified by Ni^2+^-NTA affinity chromatography followed by ion-exchange chromatography as described elsewhere (*29*, *31*). The stabilized Q-SNARE complex was composed of syntaxin-1A (amino acids 183 to 288) and SNAP-25A (no cysteine, cysteines replaced by alanines) in a 1:1 molar ratio by the C-terminal VAMP-2 fragment (amino acids 49–96), and was purified as described earlier (*39*). The soluble cytoplasmic domain (CD) of the stabilized Q-SNARE complex with syntaxin-1A (amino acids 183 to 262) was purified as described earlier (*39*). The soluble cytoplasmic region of VAMP-2 (amino acids 1 to 96) and the C2AB domain of synaptotagmin-1 (amino acids 97 to 421) were purified as described previously (*68*). The stabilized Q-SNARE complexes were purified by Ni^2+^-NTA affinity chromatography followed by ion-exchange chromatography on a Mono Q column (GE Healthcare, Piscataway, NJ) in the presence of 50 mM *n*-octyl-β-d-glucoside (OG) (*29*). The point-mutated C2AB domain (S342C) (C2AB-Alexa 488) (*68*) and VAMP-2 (49–96) (T79C) (*29*, *39*) in the stabilized Q-SNARE complex were labeled with Alexa Fluor 488 C5 maleimide. MARCKS ED was labeled at the N terminus with BODIPY TR-X succinimidyl ester. Protein structures were visualized with PyMOL; Protein Data Bank ID 1BYN for the C2A domain, 1K5W for the C2B domain, 3IPD for the SNARE complex, and 1CLL for CaM.

### Lipid composition of liposomes

All lipids were from Avanti Polar Lipids, unless stated otherwise; Oregon Green 488 DHPE was from Thermo Fisher Scientific. Lipid composition (molar percentages) of PM-liposomes containing the Q-SNARE complex: 45% PC (l-α-phosphatidylcholine, catalog no. 840055), 15% PE (l-α-phosphatidylethanolamine, catalog no. 840026), 10% PS (l-α-phosphatidylserine, catalog no. 840032), 25% Chol (cholesterol, catalog no. 700000), 4% PI (l-α-phosphatidylinositol, catalog no. 840042), and 1% PIP2 (catalog no. 840046). In case of removing PS/PIP2, PC contents were accordingly adjusted. For vesicle fusion lipid-mixing assays, 1.5% 1,2-dioleoyl-*sn*-glycero-3-phosphoethanolamine-*N*-(7-nitrobenz-2-oxa-1,3-diazol-4-yl) (NBD-PE, catalog no. 810145) as a donor and 1.5% 1,2-dioleoyl-*sn*-glycero-3-phosphoethanolamine-*N*-lissamine Rho B sulfonyl ammonium salt (Rho-PE, catalog no. 810150) as an acceptor dye were incorporated in PM-liposomes (accordingly 12% unlabeled PE). For FRET measurement using the C2AB domain labeled with Alexa 488, 1.5% Rho-DOPE was included in liposomes as an acceptor dye; lipid composition of liposomes: protein-free, 45% PC, 13.5% PE, 1.5% Rho-PE, 10% PS, 25% Chol, 4% PI, and 1% PIP2. For FRET measurement using MARCKS ED labeled with BODIPY TR, 1.5% Oregon Green 488 DHPE (catalog no. O12650, Thermo Fisher Scientific) was included in liposomes as a donor dye; lipid composition of liposomes: protein-free, 45% PC, 13.5% PE, 1.5% Oregon Green 488-DHPE, 10% PS, 25% Chol, 4% PI, and 1% PIP2.

### Preparation of proteoliposomes

Incorporation of the stabilized Q-SNARE complex into large unilamellar vesicles (LUVs) was achieved by OG-mediated reconstitution, called the direct method, i.e., incorporation of proteins into preformed liposomes (*12*, *13*, *29*, *31*). LUVs prepared by the direct method were used, unless stated otherwise. Briefly, lipids dissolved in chloroform were mixed according to lipid composition. The solvent was removed using a dry nitrogen stream in a fume hood to form a lipid film, and then lipids were resuspended in 0.5 ml of buffer containing 150 mM KCl and 20 mM Hepes-KOH, pH 7.4. After sonication on ice, multilamellar vesicles were extruded using polycarbonate membranes of pore size 100 nm (Avanti Polar lipids) to give uniformly distributed LUVs with an average diameter of 110 nm (*13*). After the preformed LUVs had been prepared, SNARE proteins (for PM-liposomes) were incorporated into liposomes by using OG, a mild nonionic detergent, then OG was removed by dialysis overnight in 1 liter buffer containing 150 mM KCl and 20 mM Hepes-KOH, pH 7.4, together with 2 g of SM-2 adsorbent beads. The protein-to-lipid ratio in proteoliposomes was 1:500 (*n*/*n*).

### Vesicle fusion assay

A FRET-based lipid-mixing assay was performed to monitor native vesicle fusion in vitro (*13*, *29*, *31*–*33*). LDCV and SV fusion assays were performed at 37°C in 1 ml of fusion buffer containing 120 mM K-glutamate, 20 mM K-acetate, 20 mM Hepes-KOH (pH 7.4), 1 mM MgCl2, and 3 mM ATP. ATP should be made freshly before experiments because it is easily destroyed by freezing and thawing. Free Ca^2+^ concentration in the presence of ATP and Mg^2+^ was calibrated using the MaxChelator simulation program. The fluorescence dequenching signal was measured using Fluoromax (Horiba Jobin Yvon) with wavelengths of 460 nm for excitation (Ex) and 538 nm for emission (Em). Fluorescence values were normalized as a percentage of maximum donor fluorescence (total fluorescence) after the addition of 0.1% Triton X-100 at the end of experiments.

### Fluorescence anisotropy measurements

Anisotropy measurement (*30*, *31*) was carried out at 37°C in 1 ml of buffer containing 120 mM K-glutamate, 20 mM K-acetate, 20 mM Hepes-KOH (pH 7.4), 1 mM MgCl2, and 3 mM ATP. Anisotropy (*r*) was calculated as *r* = (*I*VV − *G* × *I*VH)/(*I*VV + 2 × *G* × *I*VH), where *I*VV denotes the fluorescence intensity with vertically polarized excitation and vertical polarization on the detected emission, and *I*VH denotes the fluorescence intensity when using a vertical polarizer on the excitation and horizontal polarizer on the emission. *G* is a grating factor used as a correction for the instrument’s differential transmission of the two orthogonal vector orientations. Lipid composition of PM-liposomes (protein-free) was identical to those used in a fusion assay except labeled PE (45% PC, 15% PE, 10% PS, 25% Chol, 4% PI, and 1% PIP2). Anisotropy (in arbitrary units) was presented as *A*/*A*0, where *A*0 is the initial value. MARCKS ED labeled with BODIPY TR, Ex/Em = 590/620 nm. The SNARE complex (VAMP-2, amino acids 49 to 96) and the C2AB domain labeled with Alexa 488, Ex/Em = 488/516 nm. The protein-to-lipid ratio in proteoliposomes was 1:500 (*n*/*n*).

### Fluorescence resonance energy transfer

The C2AB domain labeled with Alexa 488 (a donor dye) was incubated with liposomes that include 1.5% Rho-DOPE (an acceptor dye); donor fluorescence signal was measured with wavelengths of 488 nm for excitation and 516 nm for emission (*13*). MARCKS ED labeled with BODIPY TR (an acceptor dye) was incubated with liposomes that incorporate 1.5% Oregon Green 488 DHPE (a donor dye); donor fluorescence signal was measured with wavelengths of 500 nm for excitation and 540 nm for emission. Unless otherwise stated, liposomes were LUVs prepared by the direct method. Donor fluorescence signal was measured at 37°C using Fluoromax (Horiba Jobin Yvon) in 1 ml of buffer containing 120 mM K-glutamate, 20 mM K-acetate, 20 mM Hepes-KOH (pH 7.4), 1 mM MgCl2, and 3 mM ATP. FRET was normalized as *F*/*F*0, where *F*0 represents the initial value of the donor fluorescence intensity.

### Preparation of bovine chromaffin cells

Chromaffin cells were isolated from the bovine adrenal gland medullae by two-step collagenase digestion as previously described (*69*, *70*). The cells were grown on poly-D-lysine–coated glass coverslips in Dulbecco’s modified Eagle’s medium/F-12 (catalog no. 11320033, Gibco) containing 10% fetal bovine serum (catalog no. SH30071, HyClone) and 1% antibiotics (catalog no. 10378016, Gibco).

### Amperometric measurement

Recordings of LDCV exocytosis from chromaffin cells were performed as described previously (*69*). Chromaffin cells were buffered with amine-free solution containing 137.5 mM NaCl, 2.5 mM KCl, 2 mM CaCl2, 1 mM MgCl2, 10 mM D-glucose, and 10 mM Hepes-NaOH (pH 7.3). Carbon-fiber electrodes were fabricated with 8-μm-diameter carbon fibers and back-filled with 3 M KCl. The amperometric current, which is generated by oxidation of catecholamine, was measured using an axopatch 200B amplifier (Axon Instruments Inc., CA) operated in a voltage-clamp mode at a holding potential of +650 mV. Amperometric signals were low-pass filtered at 1 kHz and sampled at 500 Hz. For data acquisition and analysis, pCLAMP 11 software (Axon Instruments) was used.

### Statistical analysis

Data analysis was performed using OriginPro 2019 software (OriginLab Corporation, Northampton, MA, USA) and GraphPad Prism 9 (GraphPad Software, San Diego, CA, USA). Data are means ± SD or SEM. One-way analysis of variance (ANOVA) test with Bonferroni correction was used to determine any statistically significant differences among three or more independent groups. Probabilities *P* < 0.05 were considered significant.
