# Supplementary material for: Ca2+/calmodulin and protein kinase C (PKC) reverse the vesicle fusion arrest by unmasking PIP2
Source: Sci Adv. 2025 Feb 26;11(9):eadr9859. doi: 10.1126/sciadv.adr9859 (PMC11864169; doi:10.1126/sciadv.adr9859)
Supplement: Supplementary file 1 — Figs. S1 to S3 Legend for dataset S1 [file sciadv.adr9859_sm.pdf]

Science Advances
AAAS

Supplementary Materials for

Ca^{2+}/calmodulin and protein kinase C (PKC) reverse the vesicle fusion arrest by unmasking PIP_2

Houda Yasmine Ali Moussa *et al.*

Corresponding author: Yongsoo Park, ypark@hbku.edu.qa

Sci. Adv. **11**, eadr9859 (2025)
DOI: 10.1126/sciadv.adr9859

The PDF file includes:

Figs. S1 to S3
Legend for dataset S1

Other Supplementary Material for this manuscript includes the following:

Dataset S1

Fig. S1

Interaction of MACRKS ED with CaM. (**A**) Monitoring the interaction of MARCKS ED with CaM using anisotropy. ED binds to CaM and PS/PIP_2-containing liposomes. MARCKS ED was labeled with BODIPY TR. Lipid composition of liposomes (protein-free): 45% PC, 15% PE, 10% PS, 25% Chol, 4% PI, and 1% PIP_2. When PS and PIP_2 were removed (no PS/PIP_2), PC contents were adjusted accordingly. Ca^{2+} was omitted.

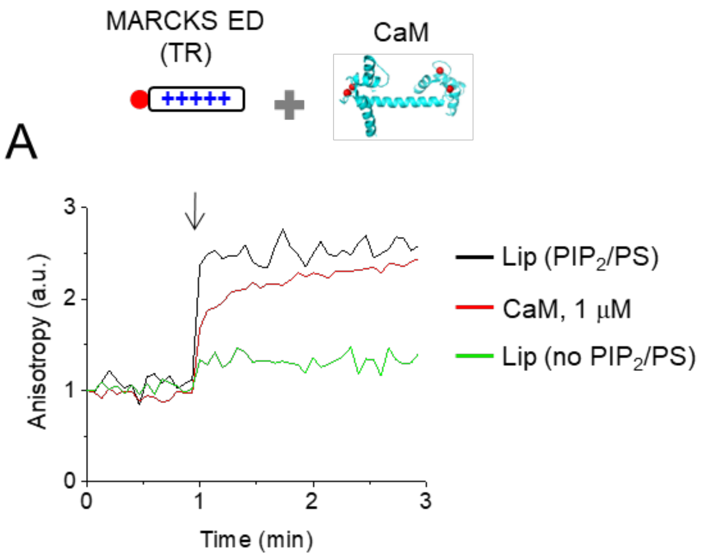

Fig. S2

No effect of CaM on Ca^{2+}-dependent C2AB membrane binding. (**A**) FRET measurement was applied to monitor the membrane binding of the C2AB domain as described in **Fig. 2C**. The C2AB domain (Syt-$1_{97\text{-}421}$) was labeled with Alexa Fluor 488 at S342C (green dot) as a donor dye, and liposomes (Lip.) incorporated Rhodamine (Rho)-PE (red) as an acceptor dye. Lipid composition of liposomes for FRET: protein-free, 45% PC, 13.5% PE, 1.5% Rho-PE, 10% PS, 25% Chol, 4% PI, and 1% PIP_2.

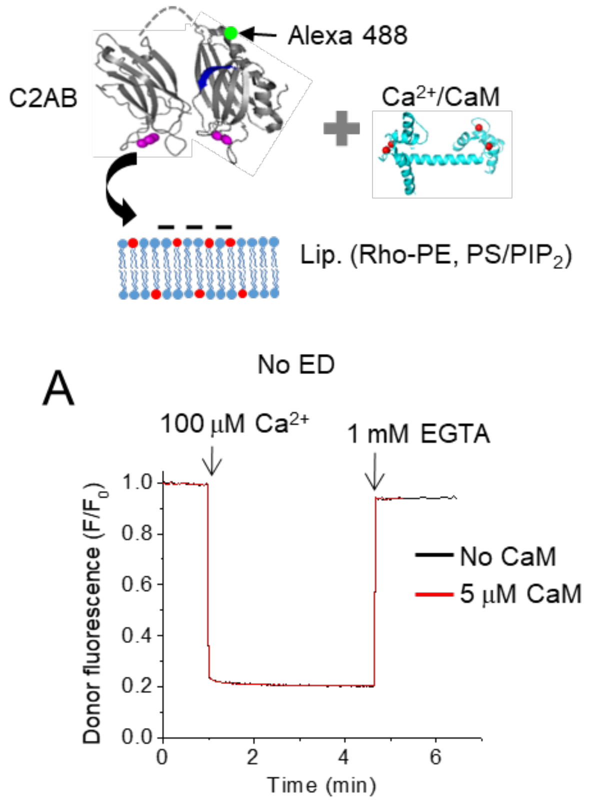

Fig. S3

No effect of CaM on Ca^{2+}-dependent SV fusion. (**A**) SV fusion with PM-liposomes using a lipid-mixing assay. Addition of 100 μM free Ca^{2+} accelerated SV fusion, but 5 μM CaM alone had no effect on Ca^{2+}-dependent SV fusion. Physiological ionic strength with 1 mM $MgCl_2$/3 mM ATP was used. Data are means ± SD from 3 independent experiments.

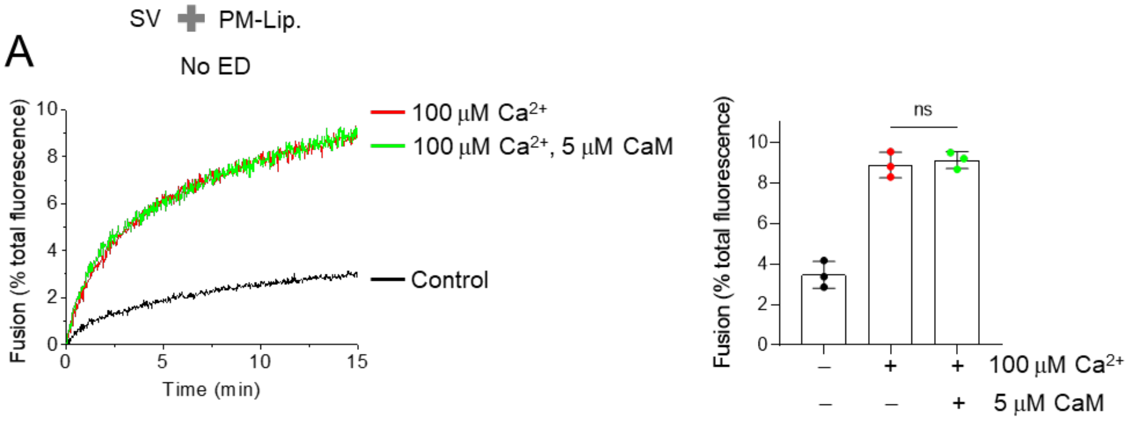

Dataset of all figures
